# 3-Amino-1-(2*H*-1,3-benzodioxol-5-yl)-9,10-dihydro­phenanthrene-2,4-dicarbonitrile

**DOI:** 10.1107/S1600536811033563

**Published:** 2011-08-27

**Authors:** Abdullah M. Asiri, Abdulrahman O. Al-Youbi, Hassan M. Faidallah, Seik Weng Ng, Edward R. T. Tiekink

**Affiliations:** a Chemistry Department, Faculty of Science, King Abdulaziz University, PO Box 80203, Jeddah, Saudi Arabia; b The Center of Excellence for Advanced Materials Research, King Abdulaziz University, PO Box 80203, Jeddah, Saudi Arabia; c Department of Chemistry, University of Malaya, 50603 Kuala Lumpur, Malaysia

## Abstract

In the title compound, C_23_H_15_N_3_O_2_, significant deviations from planarity are evidenced in the values of the dihedral angles formed between the amino-benzene ring and the benzene rings of the 1,3-benzodioxole [65.38 (12)°] and 1,2-dihydro­naphthalene [26.27 (14)°] residues; the dioxole ring has an envelope conformation with the methyl­ene-C being the flap atom. The amino-H atoms form hydrogen bonds to one of the dioxole-O atoms and to one of the cyano-N atoms to generate a two-dimensional array with a zigzag topology that stacks along the (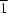 0 2) plane.

## Related literature

For background to the biological activity of related compounds, see: Aly *et al.* (1991 ▶); Al-Saadi *et al.* (2005 ▶); Rostom *et al.* (2011 ▶). For ring conformational analysis, see: Cremer & Pople (1975 ▶).
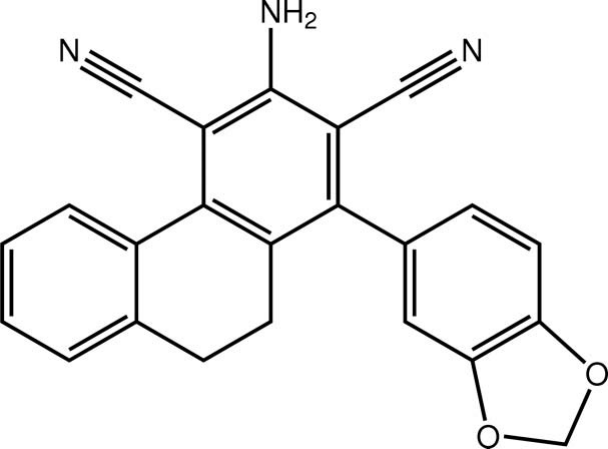


## Experimental

### 

#### Crystal data

C_23_H_15_N_3_O_2_
*M* *_r_* = 365.38
Monoclinic, 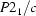

*a* = 8.9280 (6) Å
*b* = 22.4518 (13) Å
*c* = 8.9473 (6) Å
β = 109.058 (7)°
*V* = 1695.18 (19) Å^3^
*Z* = 4
Mo *K*α radiation
μ = 0.09 mm^−1^
*T* = 100 K
0.25 × 0.25 × 0.05 mm

#### Data collection

Agilent Technologies SuperNova Dual diffractometer with Atlas detector
Absorption correction: multi-scan (*CrysAlis PRO*; Agilent, 2010 ▶) *T*_min_ = 0.776, *T*_max_ = 1.000
9604 measured reflections
3775 independent reflections
2570 reflections with *I* > 2σ(*I*)
*R*_int_ = 0.042

#### Refinement

*R*[*F* ^2^ > 2σ(*F* ^2^)] = 0.065
*wR*(*F* ^2^) = 0.167
*S* = 1.02
3775 reflections
261 parameters
2 restraints
H atoms treated by a mixture of independent and constrained refinement
Δρ_max_ = 0.65 e Å^−3^
Δρ_min_ = −0.30 e Å^−3^

### 

Data collection: *CrysAlis PRO* (Agilent, 2010 ▶); cell refinement: *CrysAlis PRO*; data reduction: *CrysAlis PRO*; program(s) used to solve structure: *SHELXS97* (Sheldrick, 2008 ▶); program(s) used to refine structure: *SHELXL97* (Sheldrick, 2008 ▶); molecular graphics: *ORTEP-3* (Farrugia, 1997 ▶) and *DIAMOND* (Brandenburg, 2006 ▶); software used to prepare material for publication: *publCIF* (Westrip, 2010 ▶).

## Supplementary Material

Crystal structure: contains datablock(s) global, I. DOI: 10.1107/S1600536811033563/om2463sup1.cif

Structure factors: contains datablock(s) I. DOI: 10.1107/S1600536811033563/om2463Isup2.hkl

Supplementary material file. DOI: 10.1107/S1600536811033563/om2463Isup3.cml

Additional supplementary materials:  crystallographic information; 3D view; checkCIF report

## Figures and Tables

**Table 1:** Hydrogen-bond geometry (Å, °)

*D*—H⋯*A*	*D*—H	H⋯*A*	*D*⋯*A*	*D*—H⋯*A*
N2—H1⋯O1^i^	0.88 (1)	2.40 (2)	3.231 (3)	157 (3)
N2—H2⋯N1^ii^	0.88 (1)	2.37 (2)	3.188 (3)	156 (3)

Symmetry codes: (i) 
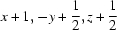
; (ii) 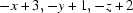.

**Fig. 1.**
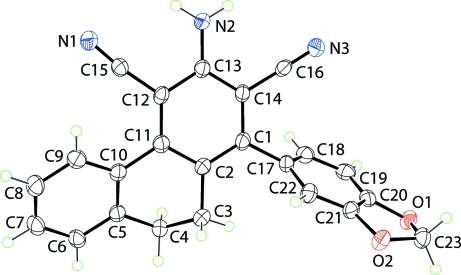
The molecular structure of (I) showing the atom-labelling scheme and displacement ellipsoids at the 50% probability level.

**Fig. 2.**
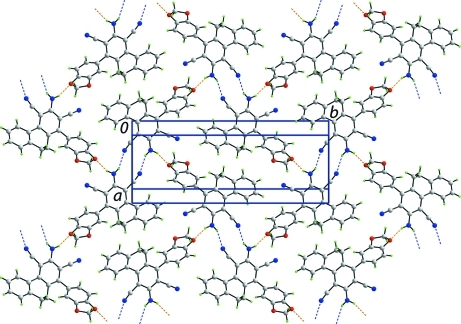
Supramolecular array in (I) viewed towards the (1 0 2) plane. The N—H···O and N—H···.N hydrogen bonds are shown as orange and blue dashed lines, respectively.

## supplementary crystallographic information

### Comment

The study of the title compound (I) was motivated by recent reports of the biological activity of related compounds (Aly *et al.*, 1991; Al-Saadi *et al.*, 2005; Rostom *et al.*, 2011).

With respect to the amino-benzene ring, the benzene rings of the 1,3-benzodioxole and 1,2-dihydronaphthalene residues form dihedral angles of 65.38 (12) and 26.27 (14) °, respectively, indicating non-planarity in the molecule. The five-membered dioxole ring has an envelope conformation with the methylene-C23 atom being the flap atom. The Cremer & Pople (1975) parameters defining the five-membered ring are q_2_ = 0.182 (3) Å and φ_2_ = 324.8 (9) Å. In the 1,2-dihydronaphthalene residue, the cyclohexa-1,3-diene ring has a distorted half-chair conformation as defined by the following parameters (Cremer & Pople, 1975): q_2_ = 0.503 (3) Å, φ_2_ = 265.5 (3) °, q_3_ = -0.189 (3) Å, and puckering amplitude Q = 0.537 (3) Å.

In the crystal structure, supramolecular arrays with zigzag topology and running parallel to the (1 0 2) plane are formed through N—H···O(dioxole) and *N*—*H*···*N*(cyano) hydrogen bonding Table 1 and Fig. 2.

### Experimental

A mixture of the piperonaldehyde (1.5 g,10 mmol), 1-tetralone (1.46 g, 10 mmol), ethyl cyanoacetate (1.1 g, 10 mmol) and ammonium acetate (6.2 g, 80 mmol) in absolute ethanol (50 ml) was refluxed for 6 h. The reaction mixture was allowed to cool and the precipitate that formed was filtered, washed with water, dried and recrystallized from DMF; *M*.pt.: 549–551 K.

### Refinement

Carbon-bound H-atoms were placed in calculated positions [C—H 0.95 to 0.99 Å, *U*_iso_(H) = 1.2*U*_eq_(C)] and were included in the refinement in the riding model approximation. The amino-H atoms were located in a difference Fourier map, and subsequently refined freely. The maximum and minimum residual electron density peaks of 0.65 and 0.30 e Å^-3^, respectively, were located 0.92 Å and 0.65 Å from the H19 and C23 atoms, respectively.

### Crystal data

**Table:**

C_23_H_15_N_3_O_2_	*F*(000) = 760
*M**_r_* = 365.38	*D*_x_ = 1.432 Mg m^−^^3^
Monoclinic, *P*2_1_/*c*	Mo *K*α radiation, λ = 0.71073 Å
Hall symbol: -P 2ybc	Cell parameters from 2779 reflections
*a* = 8.9280 (6) Å	θ = 2.4–29.3°
*b* = 22.4518 (13) Å	µ = 0.09 mm^−^^1^
*c* = 8.9473 (6) Å	*T* = 100 K
β = 109.058 (7)°	Plate, orange
*V* = 1695.18 (19) Å^3^	0.25 × 0.25 × 0.05 mm
*Z* = 4	

### Data collection

**Table:**

Agilent Technologies SuperNova Dual diffractometer with Atlas detector	3775 independent reflections
Radiation source: SuperNova (Mo) X-ray Source	2570 reflections with *I* > 2σ(*I*)
mirror	*R*_int_ = 0.042
Detector resolution: 10.4041 pixels mm^-1^	θ_max_ = 27.5°, θ_min_ = 2.6°
ω scan	*h* = −9→11
Absorption correction: multi-scan (*CrysAlis PRO*; Agilent, 2010)	*k* = −29→24
*T*_min_ = 0.776, *T*_max_ = 1.000	*l* = −11→11
9604 measured reflections	

### Refinement

**Table:**

Refinement on *F*^2^	Primary atom site location: structure-invariant direct methods
Least-squares matrix: full	Secondary atom site location: difference Fourier map
*R*[*F*^2^ > 2σ(*F*^2^)] = 0.065	Hydrogen site location: inferred from neighbouring sites
*wR*(*F*^2^) = 0.167	H atoms treated by a mixture of independent and constrained refinement
*S* = 1.02	*w* = 1/[σ^2^(*F*_o_^2^) + (0.0527*P*)^2^ + 2.2435*P*] where *P* = (*F*_o_^2^ + 2*F*_c_^2^)/3
3775 reflections	(Δ/σ)_max_ < 0.001
261 parameters	Δρ_max_ = 0.65 e Å^−^^3^
2 restraints	Δρ_min_ = −0.30 e Å^−^^3^

### Special details

**Table:**

Geometry. All e.s.d.'s (except the e.s.d. in the dihedral angle between two l.s. planes) are estimated using the full covariance matrix. The cell e.s.d.'s are taken into account individually in the estimation of e.s.d.'s in distances, angles and torsion angles; correlations between e.s.d.'s in cell parameters are only used when they are defined by crystal symmetry. An approximate (isotropic) treatment of cell e.s.d.'s is used for estimating e.s.d.'s involving l.s. planes.
Refinement. Refinement of *F*^2^ against ALL reflections. The weighted *R*-factor *wR* and goodness of fit *S* are based on *F*^2^, conventional *R*-factors *R* are based on *F*, with *F* set to zero for negative *F*^2^. The threshold expression of *F*^2^ > σ(*F*^2^) is used only for calculating *R*-factors(gt) *etc*. and is not relevant to the choice of reflections for refinement. *R*-factors based on *F*^2^ are statistically about twice as large as those based on *F*, and *R*- factors based on ALL data will be even larger.

### Fractional atomic coordinates and isotropic or equivalent isotropic displacement parameters (Å^2^)

**Table:**

	*x*	*y*	*z*	*U*_iso_*/*U*_eq_	
O1	0.4763 (2)	0.18869 (9)	0.8357 (2)	0.0314 (5)	
O2	0.4316 (2)	0.28084 (9)	0.9262 (2)	0.0331 (5)	
N1	1.3481 (3)	0.53871 (11)	0.9128 (3)	0.0338 (6)	
N2	1.3145 (3)	0.40625 (12)	1.0585 (3)	0.0336 (6)	
H1	1.344 (4)	0.3728 (9)	1.112 (4)	0.052 (11)*	
H2	1.389 (3)	0.4315 (12)	1.058 (4)	0.046 (10)*	
N3	1.1365 (3)	0.27041 (11)	1.0990 (3)	0.0334 (6)	
C1	0.8958 (3)	0.37338 (12)	0.8347 (3)	0.0258 (6)	
C2	0.8496 (3)	0.42563 (13)	0.7482 (3)	0.0272 (6)	
C3	0.6849 (3)	0.43271 (14)	0.6300 (4)	0.0342 (7)	
H3A	0.6205	0.4587	0.6746	0.041*	
H3B	0.6323	0.3934	0.6065	0.041*	
C4	0.6975 (3)	0.46015 (13)	0.4790 (3)	0.0306 (7)	
H4A	0.7561	0.4329	0.4310	0.037*	
H4B	0.5902	0.4663	0.4021	0.037*	
C5	0.7827 (3)	0.51885 (12)	0.5167 (3)	0.0270 (6)	
C6	0.7385 (3)	0.56661 (13)	0.4116 (4)	0.0297 (6)	
H6	0.6541	0.5618	0.3148	0.036*	
C7	0.8153 (3)	0.62062 (13)	0.4460 (4)	0.0343 (7)	
H7	0.7874	0.6523	0.3717	0.041*	
C8	0.9328 (4)	0.62819 (13)	0.5890 (4)	0.0360 (7)	
H8	0.9838	0.6657	0.6145	0.043*	
C9	0.9777 (3)	0.58157 (13)	0.6968 (4)	0.0312 (7)	
H9	1.0571	0.5879	0.7962	0.037*	
C10	0.9068 (3)	0.52541 (12)	0.6600 (3)	0.0263 (6)	
C11	0.9590 (3)	0.47229 (12)	0.7635 (3)	0.0261 (6)	
C12	1.1138 (3)	0.46557 (12)	0.8686 (3)	0.0240 (6)	
C13	1.1643 (3)	0.41274 (12)	0.9576 (3)	0.0259 (6)	
C14	1.0515 (3)	0.36702 (12)	0.9370 (3)	0.0243 (6)	
C15	1.2379 (3)	0.50862 (12)	0.8885 (3)	0.0268 (6)	
C16	1.0980 (3)	0.31309 (13)	1.0264 (3)	0.0271 (6)	
C17	0.7828 (3)	0.32335 (13)	0.8240 (3)	0.0260 (6)	
C18	0.8070 (3)	0.26822 (13)	0.7666 (3)	0.0304 (6)	
H18	0.8925	0.2635	0.7265	0.037*	
C19	0.7098 (3)	0.21922 (14)	0.7657 (3)	0.0315 (7)	
H19	0.7270	0.1814	0.7264	0.038*	
C20	0.5905 (3)	0.22874 (13)	0.8238 (3)	0.0274 (6)	
C21	0.5615 (3)	0.28346 (13)	0.8782 (3)	0.0273 (6)	
C22	0.6548 (3)	0.33250 (13)	0.8791 (3)	0.0278 (6)	
H22	0.6335	0.3703	0.9151	0.033*	
C23	0.3996 (4)	0.21835 (13)	0.9305 (4)	0.0326 (7)	
H23A	0.4403	0.2035	1.0405	0.039*	
H23B	0.2840	0.2110	0.8889	0.039*	

### Atomic displacement parameters (Å^2^)

**Table:**

	*U*^11^	*U*^22^	*U*^33^	*U*^12^	*U*^13^	*U*^23^
O1	0.0306 (11)	0.0281 (11)	0.0333 (11)	−0.0128 (9)	0.0074 (8)	−0.0032 (9)
O2	0.0300 (11)	0.0322 (12)	0.0402 (12)	−0.0058 (9)	0.0157 (9)	−0.0018 (9)
N1	0.0301 (13)	0.0236 (13)	0.0451 (15)	−0.0017 (11)	0.0086 (11)	0.0032 (11)
N2	0.0231 (12)	0.0274 (15)	0.0453 (15)	−0.0042 (11)	0.0046 (11)	0.0105 (12)
N3	0.0318 (13)	0.0273 (14)	0.0369 (14)	−0.0058 (11)	0.0051 (11)	0.0039 (12)
C1	0.0254 (14)	0.0258 (15)	0.0292 (14)	−0.0026 (11)	0.0131 (11)	0.0030 (12)
C2	0.0226 (13)	0.0283 (16)	0.0325 (15)	−0.0007 (11)	0.0115 (11)	0.0044 (12)
C3	0.0238 (14)	0.0345 (18)	0.0443 (18)	−0.0021 (13)	0.0112 (13)	0.0090 (14)
C4	0.0269 (14)	0.0267 (16)	0.0378 (16)	−0.0010 (12)	0.0100 (12)	0.0064 (13)
C5	0.0237 (14)	0.0248 (15)	0.0378 (16)	0.0040 (11)	0.0171 (12)	0.0048 (12)
C6	0.0289 (15)	0.0259 (16)	0.0384 (16)	0.0069 (12)	0.0166 (12)	0.0063 (13)
C7	0.0349 (16)	0.0234 (16)	0.0483 (19)	0.0092 (13)	0.0187 (14)	0.0068 (14)
C8	0.0350 (16)	0.0185 (15)	0.057 (2)	0.0030 (12)	0.0178 (15)	0.0002 (14)
C9	0.0273 (14)	0.0246 (16)	0.0434 (17)	0.0053 (12)	0.0141 (13)	0.0003 (13)
C10	0.0219 (13)	0.0221 (15)	0.0397 (16)	0.0035 (11)	0.0165 (12)	0.0021 (12)
C11	0.0257 (14)	0.0245 (15)	0.0313 (15)	0.0018 (11)	0.0134 (11)	0.0017 (12)
C12	0.0232 (13)	0.0193 (14)	0.0339 (15)	−0.0005 (11)	0.0151 (11)	−0.0012 (12)
C13	0.0256 (14)	0.0218 (15)	0.0317 (15)	−0.0014 (11)	0.0112 (11)	0.0008 (12)
C14	0.0252 (14)	0.0209 (14)	0.0288 (14)	−0.0014 (11)	0.0114 (11)	0.0024 (11)
C15	0.0271 (14)	0.0217 (15)	0.0317 (15)	0.0023 (12)	0.0096 (11)	0.0023 (12)
C16	0.0211 (13)	0.0292 (16)	0.0306 (15)	−0.0066 (12)	0.0081 (11)	−0.0009 (13)
C17	0.0257 (14)	0.0275 (15)	0.0240 (14)	−0.0042 (12)	0.0071 (11)	0.0043 (12)
C18	0.0275 (14)	0.0352 (17)	0.0271 (15)	−0.0003 (13)	0.0070 (11)	0.0019 (13)
C19	0.0290 (15)	0.0331 (17)	0.0272 (15)	0.0010 (13)	0.0022 (12)	−0.0021 (13)
C20	0.0294 (14)	0.0259 (15)	0.0210 (13)	−0.0054 (12)	0.0002 (11)	0.0015 (11)
C21	0.0251 (14)	0.0363 (17)	0.0213 (13)	−0.0030 (12)	0.0088 (11)	0.0035 (12)
C22	0.0315 (15)	0.0250 (15)	0.0279 (14)	−0.0057 (12)	0.0109 (11)	−0.0025 (12)
C23	0.0349 (16)	0.0278 (17)	0.0344 (16)	−0.0083 (13)	0.0104 (13)	0.0011 (13)

### Geometric parameters (Å, °)

**Table:**

O1—C20	1.389 (3)	C7—C8	1.374 (4)
O1—C23	1.418 (3)	C7—H7	0.9500
O2—C21	1.364 (3)	C8—C9	1.391 (4)
O2—C23	1.435 (3)	C8—H8	0.9500
N1—C15	1.154 (4)	C9—C10	1.401 (4)
N2—C13	1.358 (4)	C9—H9	0.9500
N2—H1	0.883 (10)	C10—C11	1.488 (4)
N2—H2	0.877 (10)	C11—C12	1.403 (4)
N3—C16	1.145 (4)	C12—C13	1.418 (4)
C1—C2	1.392 (4)	C12—C15	1.437 (4)
C1—C14	1.400 (4)	C13—C14	1.407 (4)
C1—C17	1.492 (4)	C14—C16	1.436 (4)
C2—C11	1.409 (4)	C17—C18	1.384 (4)
C2—C3	1.513 (4)	C17—C22	1.399 (4)
C3—C4	1.522 (4)	C18—C19	1.400 (4)
C3—H3A	0.9900	C18—H18	0.9500
C3—H3B	0.9900	C19—C20	1.346 (4)
C4—C5	1.504 (4)	C19—H19	0.9500
C4—H4A	0.9900	C20—C21	1.376 (4)
C4—H4B	0.9900	C21—C22	1.379 (4)
C5—C6	1.396 (4)	C22—H22	0.9500
C5—C10	1.402 (4)	C23—H23A	0.9900
C6—C7	1.378 (4)	C23—H23B	0.9900
C6—H6	0.9500		
			
C20—O1—C23	104.5 (2)	C5—C10—C11	118.6 (2)
C21—O2—C23	104.3 (2)	C12—C11—C2	119.0 (2)
C13—N2—H1	120 (2)	C12—C11—C10	122.9 (2)
C13—N2—H2	121 (2)	C2—C11—C10	118.0 (2)
H1—N2—H2	117 (3)	C11—C12—C13	122.0 (2)
C2—C1—C14	120.1 (2)	C11—C12—C15	124.2 (2)
C2—C1—C17	121.8 (2)	C13—C12—C15	113.7 (2)
C14—C1—C17	118.1 (2)	N2—C13—C14	121.2 (3)
C1—C2—C11	120.2 (3)	N2—C13—C12	121.8 (2)
C1—C2—C3	121.4 (2)	C14—C13—C12	117.0 (2)
C11—C2—C3	118.3 (2)	C1—C14—C13	121.8 (3)
C2—C3—C4	109.1 (2)	C1—C14—C16	119.7 (2)
C2—C3—H3A	109.9	C13—C14—C16	118.5 (2)
C4—C3—H3A	109.9	N1—C15—C12	173.0 (3)
C2—C3—H3B	109.9	N3—C16—C14	179.2 (3)
C4—C3—H3B	109.9	C18—C17—C22	120.4 (3)
H3A—C3—H3B	108.3	C18—C17—C1	120.9 (2)
C5—C4—C3	109.5 (2)	C22—C17—C1	118.7 (3)
C5—C4—H4A	109.8	C17—C18—C19	122.3 (3)
C3—C4—H4A	109.8	C17—C18—H18	118.9
C5—C4—H4B	109.8	C19—C18—H18	118.9
C3—C4—H4B	109.8	C20—C19—C18	116.1 (3)
H4A—C4—H4B	108.2	C20—C19—H19	121.9
C6—C5—C10	120.1 (3)	C18—C19—H19	121.9
C6—C5—C4	120.7 (3)	C19—C20—C21	122.8 (3)
C10—C5—C4	119.2 (2)	C19—C20—O1	128.3 (3)
C7—C6—C5	121.0 (3)	C21—C20—O1	108.9 (2)
C7—C6—H6	119.5	O2—C21—C20	110.5 (2)
C5—C6—H6	119.5	O2—C21—C22	127.4 (3)
C8—C7—C6	119.3 (3)	C20—C21—C22	122.1 (2)
C8—C7—H7	120.3	C21—C22—C17	116.2 (3)
C6—C7—H7	120.3	C21—C22—H22	121.9
C7—C8—C9	120.8 (3)	C17—C22—H22	121.9
C7—C8—H8	119.6	O1—C23—O2	107.7 (2)
C9—C8—H8	119.6	O1—C23—H23A	110.2
C8—C9—C10	120.6 (3)	O2—C23—H23A	110.2
C8—C9—H9	119.7	O1—C23—H23B	110.2
C10—C9—H9	119.7	O2—C23—H23B	110.2
C9—C10—C5	118.1 (3)	H23A—C23—H23B	108.5
C9—C10—C11	123.3 (3)		
			
C14—C1—C2—C11	−0.1 (4)	C11—C12—C13—C14	−0.5 (4)
C17—C1—C2—C11	178.9 (2)	C15—C12—C13—C14	−176.4 (2)
C14—C1—C2—C3	176.7 (3)	C2—C1—C14—C13	0.9 (4)
C17—C1—C2—C3	−4.3 (4)	C17—C1—C14—C13	−178.1 (2)
C1—C2—C3—C4	−135.0 (3)	C2—C1—C14—C16	179.7 (3)
C11—C2—C3—C4	41.8 (4)	C17—C1—C14—C16	0.7 (4)
C2—C3—C4—C5	−57.5 (3)	N2—C13—C14—C1	179.3 (3)
C3—C4—C5—C6	−143.6 (3)	C12—C13—C14—C1	−0.6 (4)
C3—C4—C5—C10	35.8 (3)	N2—C13—C14—C16	0.5 (4)
C10—C5—C6—C7	0.1 (4)	C12—C13—C14—C16	−179.4 (2)
C4—C5—C6—C7	179.5 (2)	C2—C1—C17—C18	117.0 (3)
C5—C6—C7—C8	−2.8 (4)	C14—C1—C17—C18	−64.0 (4)
C6—C7—C8—C9	1.9 (4)	C2—C1—C17—C22	−65.5 (4)
C7—C8—C9—C10	1.7 (4)	C14—C1—C17—C22	113.5 (3)
C8—C9—C10—C5	−4.2 (4)	C22—C17—C18—C19	−2.1 (4)
C8—C9—C10—C11	174.4 (3)	C1—C17—C18—C19	175.3 (3)
C6—C5—C10—C9	3.4 (4)	C17—C18—C19—C20	0.1 (4)
C4—C5—C10—C9	−176.1 (2)	C18—C19—C20—C21	1.5 (4)
C6—C5—C10—C11	−175.3 (2)	C18—C19—C20—O1	179.3 (2)
C4—C5—C10—C11	5.2 (4)	C23—O1—C20—C19	169.6 (3)
C1—C2—C11—C12	−1.0 (4)	C23—O1—C20—C21	−12.3 (3)
C3—C2—C11—C12	−177.8 (3)	C23—O2—C21—C20	11.9 (3)
C1—C2—C11—C10	175.7 (2)	C23—O2—C21—C22	−168.8 (3)
C3—C2—C11—C10	−1.1 (4)	C19—C20—C21—O2	178.4 (2)
C9—C10—C11—C12	−26.2 (4)	O1—C20—C21—O2	0.2 (3)
C5—C10—C11—C12	152.4 (3)	C19—C20—C21—C22	−1.0 (4)
C9—C10—C11—C2	157.2 (3)	O1—C20—C21—C22	−179.2 (2)
C5—C10—C11—C2	−24.2 (4)	O2—C21—C22—C17	179.7 (3)
C2—C11—C12—C13	1.3 (4)	C20—C21—C22—C17	−1.0 (4)
C10—C11—C12—C13	−175.2 (2)	C18—C17—C22—C21	2.5 (4)
C2—C11—C12—C15	176.7 (3)	C1—C17—C22—C21	−174.9 (2)
C10—C11—C12—C15	0.2 (4)	C20—O1—C23—O2	19.6 (3)
C11—C12—C13—N2	179.6 (3)	C21—O2—C23—O1	−19.5 (3)
C15—C12—C13—N2	3.7 (4)		

### Hydrogen-bond geometry (Å, °)

**Table:**

*D*—H···*A*	*D*—H	H···*A*	*D*···*A*	*D*—H···*A*
N2—H1···O1^i^	0.88 (1)	2.40 (2)	3.231 (3)	157 (3)
N2—H2···N1^ii^	0.88 (1)	2.37 (2)	3.188 (3)	156 (3)

Symmetry codes: (i) *x*+1, −*y*+1/2, *z*+1/2; (ii) −*x*+3, −*y*+1, −*z*+2.
